# From one birth to the next: how cesarean section affects maternal physical activity and health in later pregnancy—A prospective cohort study

**DOI:** 10.3389/fpubh.2025.1657089

**Published:** 2025-08-13

**Authors:** Anna Weronika Szablewska, Bartosz Zając, Rita Santos-Rocha

**Affiliations:** ^1^Department of Obstetric and Gynaecological Nursing, Institute of Nursing and Midwifery, Faculty of Health Sciences with the Institute of Maritime and Tropical Medicine, Medical University of Gdańsk, Gdańsk, Poland; ^2^Laboratory of Functional Diagnostics, Central Scientific and Research Laboratory, University of Physical Culture in Krakow, Kraków, Poland; ^3^Department of Physical Activity and Health, Sport Sciences School of Rio Maior (ESDRM), Polytechnic University of Santarém, Rio Maior, Portugal; ^4^Sport Physical Activity and Health Research & Innovation Centre (SPRINT), Rio Maior, Portugal

**Keywords:** cesarean section, physical activity, pregnancy, gestational weight gain, women's health

## Abstract

**Background:**

Cesarean section (CS) is a common surgical procedure in obstetrics, and its prevalence has been increasing globally. While the immediate outcomes of CS are well-documented, its long-term effects, particularly on maternal health, remain an area of active research. One of the critical concerns is the impact of a previous CS on gestational body mass gain (GBMG), physical activity (PA) and the likelihood of undergoing another CS in subsequent pregnancies.

**Objective:**

The aim of this study is to evaluate the potential association of a previous CS on GBMG, PA levels and the likelihood of repeat cesarean delivery in a cohort of multiparous women.

**Methods:**

This prospective cohort study, enrolling 109 Caucasian women, was conducted at a tertiary care hospital in northern Poland. Participants were recruited from antenatal outpatient clinics. The participants were divided into two groups: those who underwent previous CS and those who had vaginal delivery. Data collection was conducted in two phases. In the first phase, socio-demographic information was gathered, and participants were asked to complete the Polish version of the Get Active Questionnaire for Pregnancy. In the second phase, biomedical data routinely collected during childbirth were obtained.

**Results:**

Women with a history of CS were found to have a significantly higher likelihood of excessive gestational GBMG and were more likely to undergo another cesarean delivery in subsequent pregnancies. However, no significant differences were observed between groups in terms of insufficient GBMG or PA levels before and during pregnancy.

**Conclusions:**

The results allow to suggest that previous CS is associated with an increased risk of excessive GBMG and repeat cesarean delivery. However, it does not appear to have direct impact on PA levels during pregnancy. These findings emphasize the importance of monitoring GBMG and promoting healthy lifestyle behaviors to improve maternal and outcomes, particularly in women with a history of CS. Future research is needed to explore the long-term effects of CS on maternal health and its influence on subsequent pregnancies.

## 1 Introduction

Cesarean section (CS) is a surgical procedure involving incisions through the abdominal wall and uterus to deliver a baby (1). Historically, it was performed only in life-threatening situations; however, advancements in surgical techniques and anesthesia have made it a common obstetric practice (2–4). According to the guidelines of the Polish Society of Gynecologists and Obstetricians (5) and the American College of Obstetricians and Gynecologists (3), the main indications for CS include: obstructed labor, abnormal fetal presentation, multiple pregnancy (especially when the first fetus is not in cephalic presentation), placenta previa, placental abruption, a history of uterine surgery (e.g., previous CS or myomectomy), abnormal fetal heart rate patterns (suggesting fetal distress), maternal infections such as active genital herpes and serious maternal medical conditions, i.e., severe preeclampsia or eclampsia. An analysis of data from over nine million births across 28 European countries confirms that CS is a widely used method of childbirth. In 2015, national CS rates ranged from 16.0% to 55.9% and from 16.0% to 52.2% and in 2019 (6).

The indications for performing CS are evolving and now, in many countries, they also include psychosocial factors such as fear of childbirth or even CS on maternal request, but only in the absence of medical indications. Data on the prevalence of CS upon maternal request in developed countries are limited, as many healthcare systems do not record these cases separately (7). Nevertheless, the reasons behind increasingly liberal attitudes toward CS are complex and not always simple to define. The rising number of such procedures is mainly attributed to sociocultural and economic factors as well as the widespread belief that CS is a safe and convenient alternative to vaginal birth. Advances in surgical care have further reinforced this perception, despite the fact that operative delivery still carries significant risks for both mother and child. The growing demand for CS without medical indications continues to generate controversy among clinicians and researchers due to well-documented negative health consequences in both the short- and long-term (8). The main short-term maternal complications include, among others, excessive bleeding, injury to adjacent organs (such as the bladder, intestines or ureters), uterine scar dehiscence and longer postpartum recovery (9). Long-term complications may involve bowel obstruction (ileus), abdominal wall disorders and an increased risk of complications in subsequent pregnancies, such as uterine rupture, placenta accreta spectrum disorders, ectopic pregnancy, intrauterine fetal demise and pre-term birth. In the most severe cases, additional surgical interventions may be required, for instance hysterectomy or revision of the surgical wound (10). In conclusion, although CS remains an essential tool in modern obstetrics, alike any other medical intervention, it should be used only when medically justified.

Previous research on CS has been primarily focused on short-term clinical outcomes, such as perioperative complications, neonatal condition and postpartum recovery. Significantly less attention has been given to the potential long-term impact of CS on women's health behaviors, including PA levels and body mass management—factors that are essential determinants of maternal health. It was hypothesized that women with a history of CS are more likely to undergo repeat cesarean deliveries, have lower levels of PA and are at greater risk of excessive gestational body mass gain (GMBG) or postpartum body mass retention compared to women with a history of vaginal birth. We also assumed that the decline in PA following CS is not merely a temporary phenomenon, but may persist over time and negatively affect maternal health as well as the course of future pregnancies.

## 2 Material and methods

### 2.1. Study design

The prospective cohort study was conducted at a tertiary care hospital in northern Poland, with participants recruited from patients attending the antenatal outpatient clinic. Data collection was carried out in two stages. In the first stage, women who met the inclusion criteria were invited to complete a questionnaire consisting of self-reported items presented in the form of a medical interview. Based on the information obtained during the interview, participants were categorized into two groups: those who gave birth via CS and those who had a vaginal delivery. During the second stage, access was obtained to standard biomedical data routinely collected during childbirth. All procedures adhered to the principles of the Declaration of Helsinki (1964) and its subsequent amendments. Ethical approval for the study was granted by the Bioethics Committee of the Medical University of Gdansk (Approval No. NKBBN/406-1/2024).

### 2.2. Participants

The study comprised 109 Caucasian women who met the following eligibility criteria: entry into the third trimester (after the 28th week of gestation), age of 18 years or older, fluency in Polish, absence of medical contraindications to PA, a history of at least one previous pregnancy (multiparous), no diagnosis of placenta previa and a planned delivery at a hospital associated with the outpatient clinic where recruitment took place. Participants were thoroughly informed about the purpose and protocol of the study, as well as their right to withdraw from the study at any time. The participants provided written informed consent to participate in the research. The women gave their consent not only to take part in the study but also to allow access to anonymized medical data concerning themselves. The basic characteristics of study participants are presented in Table 1.

**Table 1:** Basic characteristics of the study participants.

	**Group**		***p*-value**
	**CS**, ***n*** = **39**	**VD**, ***n*** = **80**	
Age [years]	33 (29–36)	32 (30–36)	0.860
Body height [m]	1.64 (1.60–170)	1.68 (1.63–1.70)	0.099
Pre-pregnancy body mass [kg]	65.0 (56.0–83.0)	66.5 (58.0–79.0)	0.543
Pre-pregnancy BMI [kg × m^−2^]	23.6 (20.6–31.1)	24.0 (21.1–28.0)	0.869
**Delivery mode**			
Vaginal	8 (20.5)	67 (83.7)	< 0.001^*^
Cesarean	31 (79.5)	13 (16.3)		
**Pre-pregnancy physical activity status** ^&^			
Physically active	11 (45.8)	25 (38.5)	0.530
Physically inactive	13 (54.2)	40 (61.5)		
**Physical activity status during pregnancy** ^&^			
Physically active	2 (8.7)	12 (18.5)	0.271
Physically inactive	21 (91.3)	53 (81.5)		
**Education level**			
Primary	8 (20.5)	8 (10.0)	0.274
Secondary	6 (15.4)	16 (20.0)		
Higher	25 (64.1)	56 (70.0)		
**Place of residence**			
Village	7 (18.0)	17 (21.3)	0.570
City ≤ 500k inhabitants	30 (76.9)	55 (68.7)		
City > 500k inhabitants	2 (5.1)	8 (10.0)		
**Economic status**			
Average	6 (15.4)	15 (18.8)	0.430
Good	33 (84.6)	65 (81.2)		
**Marital status**			
Single	6 (15.4)	16 (20.0)	0.637
Married	33 (84.6)	63 (78.8)		
Widowed	0 (0.0)	1 (1.2)		
Divorced	0 (0.0)	0 (0.0)		

Data are expressed as mean ± SD or *n* (%). Data do not sum up to the total sample size due to missing values. CS, cesarean section group; VD, vaginal delivery group; *p*-value, probability of obtaining the observed results under the null hypothesis; ^*^statistically significant difference. ^∧^Body mass gain during pregnancy based on pre-pregnancy BMI was assessed according to IOM recommendations for optimal maternal and fetal outcomes (31). ^&^Women classified as physically active were those who reported engaging in at least 150 min of moderate-intensity activity, 75 min of vigorous-intensity activity, or an equivalent combination per week (32).

### 2.3. Outcomes

Socio-demographic data were obtained during the medical interview. Information on PA levels was collected using the Polish version of the Get Active Questionnaire for Pregnancy (11). The Get Active Questionnaire for Pregnancy (GAQ-P) allows assessment of potential contraindications to physical activity as well as evaluation of the frequency, intensity, duration and type of physical activity undertaken before and during pregnancy, as well as the activity planned until the end of pregnancy. Data regarding GBMG and mode of delivery were extracted from standard medical records routinely collected during childbirth.

### 2.4. Statistical analysis

All statistical analyses were carried out in Python 3.13.1 using the pandas, NumPy and statsmodels.formula.api libraries. To assess the effect of delivery mode on dependent variables, logistic regression analysis was applied. For ratio-scale variables, group differences were examined using the Student's *t*-test, Welch's *t*-test or the Mann–Whitney *U* test, depending on whether the assumptions of normality (verified via the Shapiro–Wilk test) and homogeneity of variances (assessed using Levene's test) were met. For nominal variables, comparisons between groups were performed using the χ^2^ test. A *p*-value below 0.05 was considered statistically significant.

## 3 Results

### 3.1. Basic characteristics of participants

The data presented in Table 1 show that women with a history of cesarean section (CS group) and those with a previous vaginal delivery (VD group) did not differ significantly in terms of age, height, pre-pregnancy body mass or body mass index. No statistically significant differences were observed between the groups in terms of physical activity levels (before or during pregnancy), education, place of residence, and economic or marital status. The only variable for which a statistically significant difference was demonstrated regarded the mode of delivery in the current pregnancy—women in the CS group were significantly more likely to deliver via cesarean section again.

### 3.2. Outcomes

According to the data presented in Table 2, women in the CS group had a statistically significant more than two-fold increased risk of excessive gestational body mass gain compared to those in the VD group. Additionally, the likelihood of undergoing a repeat cesarean delivery was nearly 20 times higher in the CS group, and this association was also statistically significant. In contrast, a history of CS was not significantly associated with an increased risk of insufficient gestational weight gain, nor with engagement in physical activity prior to or during pregnancy.

**Table 2:** Analysis of associations between cesarean section and maternal outcomes in subsequent pregnancy.

	**Logistic regression estimates**		***p*-value**
	**Odds ratio**	**95% CI**	
**Body mass gain during pregnancy** ^∧^			
Insufficient	0.54	0.22–1.32	0.178
Excessive	2.18	1.00–4.78	0.049^*^
Adequate (ref.)			
**Delivery mode**			
Cesarean	19.97	7.51–53.12	< 0.001^*^
Vaginal (ref.)			
**Pre-pregnancy physical activity status** ^&^			
Physically active	1.35	0.53–3.49	0.530
Physically inactive (ref.)			
**Physical activity status during pregnancy** ^&^			
Physically active	0.42	0.09–2.04	0.283
Physically inactive (ref.)			

The reference category for the variable delivery mode is vaginal delivery (coded as 0). 95% CI, 95% confidence interval; *p*-value, probability of obtaining the observed results under the null hypothesis. ^∧^Body mass gain during pregnancy based on pre-pregnancy BMI was assessed according to IOM recommendations for optimal maternal and fetal outcomes (31). ^&^Women classified as physically active were those who reported engaging in at least 150 min of moderate-intensity activity, 75 min of vigorous-intensity activity, or an equivalent combination per week (32). The symbol * denotes a statistically significant difference (*p* < 0.05).

## 4 Discussion

The aim of this study was to evaluate the long-term consequences of a previous CS on the course of subsequent pregnancies and selected lifestyle-related factors, particularly PA and GBMG. To the best of the authors' knowledge, this is one of the first studies focused on the long-term behavioral and health-related consequences of a primary cesarean delivery, going beyond the commonly studied short-term postpartum outcomes. The findings indicate that a prior CS is associated with an increased risk of excessive GBMG and a higher likelihood of undergoing a repeat cesarean delivery. However, no significant associations were found between previous cesarean section and insufficient gestational body mass gain (GBMG) or lower levels of physical activity before or during pregnancy, which is consistent with the findings of other studies in this area (12).

One possible explanation for the observed association between a history of CS and excessive GBMG in subsequent pregnancies is the potential long-term impact of surgical delivery on maternal health behaviors and metabolic adaptation. Cesarean delivery may contribute to more prolonged postpartum recovery, reduced physical functioning as well as persistent changes in abdominal and pelvic musculature which, in turn, could limit the return to regular PA and predispose to gradual body mass retention between pregnancies. The retained postpartum body mass may create a baseline for excessive body mass gain during subsequent pregnancies, especially if combined with common lifestyle factors such as reduced PA or inadequate dietary habits.

Furthermore, CS is often medically linked with maternal conditions such as obesity, gestational diabetes and hypertensive disorders, which are themselves associated with altered metabolic regulation and a higher risk of excessive GBMG (13–15). Thus, the pathway may be partly biological and partially behavioral, involving both the physical limitations resulting from a prior surgical delivery and the broader context of maternal health status. Excessive GBMG carries significant health implications for both the mother and the offspring, including an increased risk of hypertensive disorders, gestational diabetes, macrosomia, postpartum body mass retention and long-term obesity, all of which may further increase the likelihood of surgical delivery in future pregnancies (13, 16). This allows to underline the significance of preconception counseling and body mass management among women with a history of cesarean section in order to minimize the cycle of repeat surgical deliveries and metabolic complications.

Excessive GBMG following a previous CS can have substantial health consequences for the mother, both in the short- and long-term. From an obstetric perspective, excessive body mass gain is associated with an increased risk of pregnancy complications such as gestational diabetes, hypertensive disorders and labor dystocia—all of which are known to elevate the likelihood of another cesarean delivery (13, 17, 18). Moreover, higher GBMG may lead to excessive fetal growth (macrosomia), which not only complicates vaginal delivery but also increases the risk of birth injuries and necessitates operative delivery (9, 19).

Beyond delivery, excessive body mass gain during pregnancy is a strong predictor of postpartum body mass retention, which contributes to long-term maternal overweightness and obesity (20–23). This, in turn, escalates the risk of developing chronic conditions such as type 2 diabetes, cardiovascular disease and metabolic syndrome (13, 20, 21). Additionally, maternal obesity negatively influences fertility and increases the risk of complications in future pregnancies, thus, creating a self-perpetuating cycle of adverse health outcomes (15). These findings allow to highlight the importance of body mass monitoring and individualized nutritional counseling during pregnancy, particularly in women with a history of cesarean section, to reduce the likelihood of excessive body mass gain and its subsequent health burdens.

A prior CS is a well-established risk factor for repeat cesarean delivery in subsequent pregnancies, and this association is both clinical and structural. Current obstetric guidelines acknowledge that previous cesarean delivery is often a direct indication for planned repeat cesarean section, especially in the absence of conditions favorable for a trial of labor after cesarean (TOLAC) (24, 25). Although TOLAC is considered a safe and recommended option in specific circumstances, including a low-transverse uterine scar and no contraindicating obstetric factors, many women do not meet these criteria or are advised against TOLAC due to individual risk profiles.

The decision to perform a repeat cesarean is often influenced by concerns related to uterine rupture, which, while relatively rare, is associated with severe maternal and neonatal morbidity as well as mortality (26, 27). Beyond the delivery itself, undergoing multiple cesarean sections substantially increases the risk of significant complications, including excessive intraoperative bleeding, placenta previa, placenta accreta spectrum disorders and surgical injuries to the bladder or bowel. Moreover, repeated cesarean deliveries are associated with a higher likelihood of postoperative infections, thromboembolic events, longer hospitalization and chronic pelvic pain (16, 27, 28).

In the current study, a significant association was not demonstrated between a history of CS and reduced PA levels before or during a subsequent pregnancy. This finding may suggest that cesarean delivery itself does not substantially influence long-term behavioral patterns related to PA. However, it remains an open question as to what the baseline PA levels were before the previous pregnancy, as it is possible that these were already insufficient. In the present research, this aspect was not assessed and the behavioral dimension of PA was the sole focus in the context of subsequent pregnancies. Moreover, to date, the available literature primarily offers guidelines and expert recommendations on the timing, safety and benefits of returning to PA after CS, as well as suggested types of exercises (29, 30), rather than objective data on the actual activity levels in this population. This gap highlights the novelty of the trial, as there is still a lack of empirical studies in which authors would directly address the PA patterns of women following cesarean delivery.

The findings of this study have significant practical implications, particularly in the context of healthcare management for women who have undergone CS. The results suggest that a history of cesarean delivery is associated with an increased risk of excessive GBMG and a higher likelihood of repeat CS delivery in subsequent pregnancies. Therefore, it is crucial to systematically monitor the metabolic health of women post-cesarean, offering appropriate preventive strategies aimed at controlling body mass gain during pregnancy and promoting PA, which could potentially reduce these adverse outcomes. In terms of decision-making regarding the mode of delivery, the need for a more individualized approach to women who have previously undergone a CS is highlighted in this study, ensuring that each case is evaluated based on the specific risks and benefits for both the mother and child.

### 4.1 Study limitations and strengths

Despite the valuable insights provided in the current study, several limitations should be acknowledged. Firstly, the observational nature of the research limits the ability to infer causal relationships between previous CS and outcomes such as excessive GBMG or repeat cesarean delivery. Additionally, the study relied on self-reported data for PA, which may introduce recall bias and affect the accuracy of the results. Another limitation is the lack of data on participants' pre-pregnancy activity levels, which could provide a more comprehensive understanding of the factors influencing postpartum PA and future pregnancy outcomes. Furthermore, the sample was limited to a specific geographic area, which may have affected the generalizability of the findings to other populations. Lastly, in the study, the long-term impact was not assessed of cesarean delivery on maternal health beyond the immediate pregnancy period, leaving room for future research to explore the enduring effects on physical well-being and overall quality of life.

The main strength of the study is that it lies in shifting the focus from immediate medical consequences to long-term behavioral outcomes, with particular emphasis on subsequent pregnancies and overall maternal well-being. Accordingly, the aim of this study was to assess the relationship between the history of cesarean section (CS) and subsequent obstetric outcomes, physical activity (PA) patterns and maternal body mass regulation. This comprehensive approach allowed for multidimensional evaluation of the interplay between prior delivery mode, lifestyle factors and health outcomes, offering valuable insights into the long-term consequences of CS on women's health. The study's strength regards its focus on real-world data collected from a well-defined population, the use of standardized measurement tools and consideration of both medical as well as behavioral determinants, which together provide a robust basis for evidence-based recommendations in postnatal care and physical activity counseling.

## 5 Conclusion

In the present study, it is highlighted that women who have had a CS in a previous pregnancy are at a higher risk of excessive GBMS and may be more likely to have another cesarean in subsequent pregnancies. The importance of carefully considering cesarean delivery due to its potential long-term impact on maternal health is also emphasized. Future studies should be focused on understanding how pre-pregnancy PA levels influence outcomes after cesarean delivery and explore the long-term effects of cesarean births on maternal health. Additionally, further research is needed to develop targeted interventions to promote safe PA and body mass management after CS, improving overall maternal well-being in subsequent pregnancies.

## Data Availability

The original contributions presented in the study are included in the article/supplementary material, further inquiries can be directed to the corresponding author.
